# Preventive and therapeutic efficacy of a pyrazole-modified chitosan Schiff base–iron nanocomposite against *Eimeria tenella* in broiler chickens: A nanotechnology-based approach to coccidiosis control

**DOI:** 10.14202/vetworld.2025.2295-2310

**Published:** 2025-08-14

**Authors:** Safinaz J. Ashoor, Hoda A. Taha, Muslimah N. Alsulami, Amira A. Hamed, Ahmed H. Nigm

**Affiliations:** 1 Faqieh Farms Laboratories, Kingdom of Saudi Arabia; 2 Department of Zoology, Faculty of Science, Ain Shams University, Abbassia, Egypt; 3 Department of Biological Sciences, College of Science, University of Jeddah, Jeddah, Saudi Arabia; 4 Department of Chemistry, Faculty of Science, Cairo University, Giza, Egypt

**Keywords:** anticoccidial nanocomposite, broiler chicken, chitosan Schiff base, *Eimeria tenella*, histopathology, iron nanoparticles, prophylactic therapy

## Abstract

**Background and Aim:**

*Coccidiosis*, caused by *Eimeria tenella*, is a significant parasitic disease affecting poultry, resulting in severe intestinal damage and substantial economic losses. The increasing resistance to conventional anticoccidial drugs necessitates novel therapeutic strategies. This study aimed to synthesize and characterize a pyrazole-modified chitosan Schiff base–iron nanocomposite (ChSB-FeNPs) and evaluate its prophylactic and therapeutic effects against *E. tenella* in experimentally infected broiler chickens.

**Materials and Methods:**

ChSB-FeNPs were synthesized by incorporating iron nanoparticles into a pyrazole-modified chitosan Schiff base matrix and characterized using Fourier-transform infrared spectroscopy, X-ray diffraction, scanning electron microscopy, and transmission electron microscope (TEM) techniques. Sixty broiler chicks were randomly assigned to six groups: Uninfected controls, prophylactic and therapeutic ChSB-FeNPs treatments, and a standard amprolium treat-ment. Birds were infected with *E. tenella* and monitored over 28 days. Clinical signs, survival, body weight, feed conversion ratio (FCR), oocyst counts, lesion scores, liver enzyme activities (aspartate aminotransferase, alanine aminotransferase, and alanine aminotransferase), lipid profiles (low-density lipoprotein, high-density lipoprotein), and histopathological changes were assessed.

**Results:**

ChSB-FeNPs-treated groups (both prophylactic and therapeutic) showed significantly reduced oocyst output, lesion scores, liver enzyme elevations, and histopathological damage compared to infected untreated controls. Prophylactic ChSB-FeNPs treatment notably improved body weight gain and FCR, with efficacy comparable to or exceeding that of amprolium. TEM confirmed the nanocomposite size (~39.5 nm), and cytotoxicity assays demonstrated safety at 0.133 μg/mL.

**Conclusion:**

ChSB-FeNPs exhibited potent anticoccidial effects, offering both preventive and therapeutic benefits against *E. tenella* infection in broilers. This nanocomposite represents a promising, next-generation alternative to conventional anticoccidial drugs, warranting further investigation for large-scale application.

## INTRODUCTION

Coccidiosis is a prevalent parasitic disease in poultry, caused by various genera and species of sporozoan parasites, with *Eimeria* being the most clinically significant [1]. Among the different forms of avian coccidiosis, cecal coccidiosis – primarily induced by *Eimeria tenella* – is the most severe due to the extensive lesions and systemic effects associated with cecal infection [2, 3]. The disease causes significant economic losses in the global poultry industry, estimated to be in the billions of dollars annually. In addition to the primary infection, coccidial lesions predispose birds to secondary bacterial infections such as necrotic enteritis, further exacerbating production losses and increasing mortality [4, 5]. Notably, necrotic enteritis alone is responsible for global losses exceeding $6 billion, driven by increased carcass condemnations and reduced feed efficiency [6].

*E. tenella* invades the cecal epithelium of broiler chickens following ingestion of sporulated oocysts, leading to epithelial destruction, impaired nutrient absorption, dehydration, bloody diarrhea, and substantial mortality. These pathological changes negatively affect growth performance and feed conversion ratio (FCR) [7–9].

To mitigate the impact of coccidiosis, poultry producers rely heavily on routine administration of anticoccidial drugs [10]. While these chemical agents have demonstrated efficacy, their prolonged and widespread use has raised significant concerns regarding drug resistance, compromised efficacy, and increased production costs – especially in commercial farming systems [11–15]. Consequently, there is an urgent need for novel, safe, and cost-effective alternatives for the prevention and treatment of coccidiosis.

Nanotechnology presents a promising frontier in veterinary medicine, particularly in poultry health, due to its ability to deliver targeted, effective, and multifunctional interventions. In the poultry sector, nanomaterials have been utilized to improve feed quality, inhibit pathogenic microbes, and stimulate immune responses [16, 17]. Recent advances have focused on the development of biologically synthesized nanoparticles as viable alternatives to traditional pharmaceuticals, with demonstrated potential in controlling parasitic diseases [18].

Chitosan, a deacetylated derivative of chitin, is a natural polysaccharide known for its versatile biological activities, including antioxidant, anticancer, and immunomodulatory properties [19]. Chemical modification of chitosan, particularly through the formation of Schiff bases, enhances its biological activity. These derivatives are synthesized by reacting the amino groups of chitosan with aldehydes or ketones, generating compounds with enhanced antibacterial and therapeutic potential [20]. The incorporation of heterocyclic moieties, such as pyrazoles, into chitosan scaffolds further enhances its pharmacological profile, due to the antimicrobial, anticancer, and antioxidant properties of pyrazoles [21].

In support of this strategy, prior research demonstrated that dechlorinated pyrazole-imidazoline derivatives exhibited potent activity against *Trypanosoma cruzi*, with docking studies identifying cruzipain as a key target enzyme. These derivatives were shown to bind effectively to the active site of cruzipain, suggesting their potential as therapeutic candidates for Chagas disease [22]. Moreover, pyrazole derivatives have been reported to disrupt parasite homeostasis by affecting sodium ion ATPases – specifically ENA P-type and ATP4-type – suggesting a similar mechanism may be relevant for *Eimeria* spp. These compounds also inhibit iron (Fe) superoxide dismutase, thereby compromising the antioxidant defense system of the parasite and promoting oxidative damage [23].

Another study by Abdel-Latif *et al*. [24] has demonstrated the anticoccidial efficacy of chitosan against *Eimeria papillata*, highlighting its role in reducing parasite load and modulating inflammation – key factors in preserving intestinal health and enhancing disease resistance in livestock.

To further potentiate chitosan’s antiparasitic effects, iron oxide (Fe_2_O_3_) nanoparticles have been incorporated to enhance its antimicrobial and anticoccidial activity. In alignment with these findings, a pyrazole-modified chitosan Schiff base–iron nanocomposite (ChSB-FeNPs) was synthesized and proposed as a novel therapeutic agent for controlling *E. tenella* infections in poultry [25].

Despite the widespread use of anticoccidial drugs such as amprolium, increasing reports of drug resistance, residual toxicity, and reduced efficacy have highlighted the limitations of conventional coccidiosis control strategies. Although natural compounds like chitosan have shown promise in modulating immune responses and reducing *Eimeria*-induced pathology, their efficacy as standalone agents remains moderate, particularly under high parasite load. Recent advances in nanotechnology have opened new avenues for enhancing the bioavailability, stability, and biological activity of such compounds. While studies have explored the use of chitosan nanoparticles and Fe_2_O_3_ nanomaterials separately, there is a paucity of research investigating the combined application of chitosan Schiff base derivatives with Fe nanoparticles, particula- rly those modified with biologically active moieties such as pyrazoles. No published studies to date have evaluated the prophylactic and therapeutic potential of pyrazole-functionalized ChSB-FeNPs against *E. tenella* infection in broiler chickens. This gap in the literature warrants experimental investigation into the synergistic anticoccidial effects of such nanocompo-sites, their histopathological impact, and their ability to improve clinical and biochemical outcomes in poultry.

The aim of this study was to synthesize and characterize a novel ChSB-FeNPs and to evaluate its efficacy as both a therapeutic and prophylactic agent against *E. tenella* infection in experimentally challenged broiler chickens. The nanocomposite was first characterized using Fourier-transform infrared spectroscopy (FTIR), X-ray diffraction (XRD), scanning electron microscopy (SEM)/energy-dispersive X-ray (EDX), and transmission electron microscopy (TEM) to confirm its structural and morphological properties. Subsequently, its anticoccidial potential was assessed by measuring clinical signs, body weight gain, FCR, oocyst shedding, lesion scoring, and survival rate. Biochemical parameters, including liver enzymes (alanine aminotransferase [ALT], aspartate aminotransferase [AST], and gamma-glutamyl transferase [GGT]), and lipid profiles (low-density lipoprotein [LDL] and high-density lipoprotein [HDL]), were evaluated to determine the systemic health impacts. Histopathological examination of cecal tissues was conducted to assess mucosal integrity and parasite development. The study further compared the outcomes of ChSB-FeNPs-treated groups with those treated using conventional amprolium, aiming to establish the nanocomposite’s efficacy as a next-generation alternative to traditional anticoccidial therapies.

## MATERIALS AND METHODS

### Ethical approval

The study protocols concerning this study were approved by the Ain Shams University Research Ethics Committee (ASU-REC), Egypt (Ethical Committee permission No. ASU-SCI/ZOOL/2024/10/1). The key aspects of animal welfare measures included providing adequate food and water, proper housing, appropriate healthcare, and humane handling.

### Study period and location

This study was conducted for 28 days (10 February to 8 March 2024) in the Veterinary Labora- tory of Fakieh^®^ Farm in the Kingdom of Saudi Arabia.

### Chemical materials and reagents

Chitin was extracted from prawn crust and used to produce chitosan as described by Yadav *et al*. [19]. The titration technique revealed that the degree of deacetylation is 89%. The viscosity measurements indicated that the average molecular weight (1.9 × 10^5^ Da) [26].

Phosphorus oxychloride (POCl_3_) [Product No. 201170, 99%], phenyl hydrazine (C_6_H_8_N_2_) [Product No. P26252, 97%], and 3-acetyl pyridine (C_7_H_7_NO) [Product No. A21207, 98%] were purchased from Merck (Germany). Dimethylformamide (DMF) [Product No. AL1836, 99%] and acetic acid [Product No. AL5432, 80%, extra-pure grade] were purchased from Alpha Chemika in India. All additional products are analytical grade and can be used without additional purification. The reference drug amprolium (Amprol, Product #09568A, 9.6% oral solution, HuvePharma Inc., Peachtree City, Georgia, USA) was purchased from the Veterinary Center of Faqih Farms in the Kingdom of Saudi Arabia.

### Synthesis of the nanocomposite

#### Synthesis of pyrazole derivative

The production of 1-phenyl-3-(pyridine-3-yl)-1H-pyrazole-4-carbaldehyde was produced by reacting 3-acetyl pyridine with phenyl hydrazine in a 1:1 molar ratio in ethanol under reflux for 8 h, as previously described by Hamed *et al*. [27]. The resulting hydrazone (0.05 mol in 10 mL of DMF) was added dropwise to a freshly prepared Vilsmeier-Haack reagent [DMF (20 mL) with POCl_3_ (0.1 mol)] in an ice bath. The temperature was then raised to 60°C–65°C, and the mixture was stirred until the reaction was complete (often 2 h–4h), until completion (monitored by thin layer chromatography [TLC]). The reaction was then quenched in ice/water, neutralized to pH ~7 using sodium bicarbonate solution, and the final product was extracted by ethanol and recrystallized to obtain the aldehyde.

#### Synthesis of pyrazole-modified Chitosan Schiff base

Chitosan Schiff base was synthesized by dissolving 2.0 g of chitosan in 150 cm^3^ of 2.0% aqueous acetic acid and 50 cm^3^ ethanol at room temperature (25°C). Predetermined amounts of pyridine-3-carboxaldehyde (in a 1:1 molar ratio to chitosan) were added to the solution, which was stirred continuously. The mixture was left to react at 60°C with stirring for 12 h. The product was precipitated using excess ethanol and a few drops of ammonium hydroxide to adjust the pH to 10. The precipitate was filtered and washed with anhydrous ethanol. The Soxhlet apparatus was used to ensure the ultra-purification of the chitosan Schiff base product through continuous washing with fresh ethanol, removing any residual unreacted aldehydes and preventing damage to the chitosan structure [27].

#### Synthesis of Fe_2_O_3_ nanoparticles

For the preparation of Fe_2_O_3_ nanoparticles, the procedure by Norihito *et al*. [28] was applied with some modifications (Reaction mixture was irradiated for 25 min instead of 120 s (at 2450 MHz and 90 watts):

- The ferrous sulfate (FeSO_4_)·7H_2_O powder was purchased from Merck [Product No. F7002, ReagentPlus ≥99%].
- One hundred mL of 0.1M FeSO_4_·7H_2_O solution was prepared in a glass flask and irradiated in a microwave oven (at 2450 MHz and 90 watts) for 25 min.
- The color of the solution changed from bright yellow to reddish brown.
- The product was washed with deionized water and air-dried at 25°C or 24 h [28].

#### Preparation of pyrazole-modified ChSB-FeNPs

For preparation of pyrazole-modified ChSB- FeNPs, chitosan Schiff base was suspended in 3% acetic acid, and the Fe nanoparticle suspension was prepa- red using a water bath sonicator (Dumax^®^, China, model: UD50Sh-2.5LQ, ultrasonic power: 50W, 28kHz, at 25°C) in absolute ethanol for 15 min, ensuring homogeneous dispersion.

**Figure 1:**
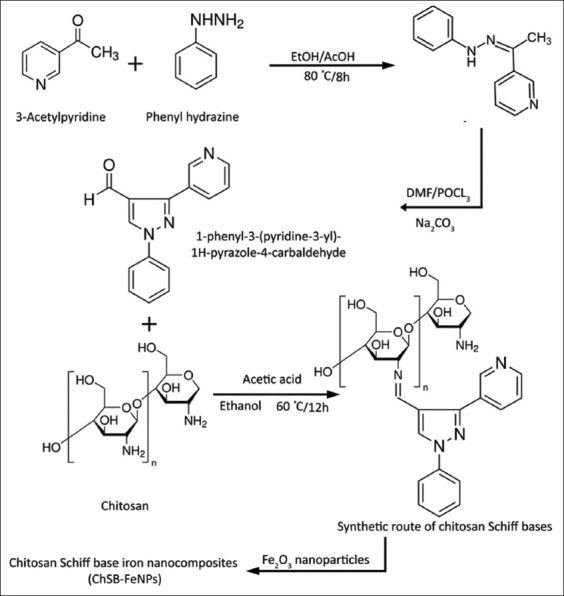
Reaction scheme for the preparation of chitosan Schiff base iron nanocomposites (ChSB-FeNPs).

The Fe nanoparticle suspension was added to the chitosan Schiff base under stirring for 1 h and then sonicated for 30 min to achieve homogenization [29] (Figure 1).

### Nanoparticle characterization (FTIR, XRD, SEM/EDX, and TEM)

The FTIR spectrum of the synthesized chitosan Schiff base derivatives was obtained using a Perkin Elmer Lambda 25 spectrophotometer (400–4000 cm^-^¹, resolution 4 cm^-^¹, Number of Scans: 60, Sample Prepa-ration: powder pressed, PerkinElmer, USA).

The sample was analyzed using a Philips X’Pert Pro MPD X-ray powder diffractometer (Netherlands) (Cu Κα radiation (Κα = 1.541 Å), 50 kV/40 mA, Scan range (2θ) = 5°–60°, Δ2θ = 0.02°, Mode of Scan: continuous).

A Quanta FEG 250 scanning electron microscope was used to examine the morphology of the synthesized product (20kV, Spot Size: 3–4, working distance: 10 mm, Detector: EBSD, Thermo Fisher Scientific FEI, 1027641, Czech Republic).

EDAX Element EDS System (20kV, Acquisition time: 120 s–300s, beam current: 1 nA–10 nA, Ametex Octane Pro, USA) was used to detect and determine the elemental composition of Fe nanoparticles in the chitosan Schiff base matrix.

A JEOL (JEM 2100, 200Kv, Mag. 20–50kX, Japan) TEM was used to verify and detect the size of the nanoparticles present in the ChSB-FeNPs matrix.

### Cytotoxicity assessment

The African green monkey kidney line (Vero) was kindly supplied by the Veterinary Serum and Vaccine Research Institute, Abasia, Cairo, Egypt, and was used for in vitro safety testing of the used materials [30]. These cells were propagated and maintained in minimum essential medium supplemented with newborn calf serum (10% for growth medium and 2% for maintenance medium). The used solvent and materials were diluted in HBSS (Hank’s Balanced Salt Solution) using a 10-fold dilution up to 1:10^7^ (Stock solution concentration of ChSB-FeNPs was 13.3 mg/mL). 25 μL of each dilution of each material was inoculated in 3 wells of a 96-well cell culture plate Corning (Falcon, USA), then 150 μL of Vero cell suspension containing 4 × 10^4^ cells/mL was added to each well.

The test included cell control without the use of any material. The plate was incubated at 37°C and examined using an inverted microscope to detect cell viability through cell deformation (from elongated-shaped cell to round one) and cell distribution (from monolayer to empty areas in the culture surface).

The LD_50_ was calculated based on the treatment of chicken groups with different concentrations of ChSB-FeNPs (50, 100, 150, and 200 μL of a 13.3 mg/mL solution).

### Animals and housing conditions

Sixty Arbor Acres broiler chicks (1 day old) were obtained from a local hatchery in Makkah, Saudi Arabia. Selected chicks were blocked by initial body weight (Average: 42 ± 2 g) before random assignment to treatment groups to ensure uniformity. No additional stratification was used. On 1 day of age, the tempera-ture was maintained until a stable temperature of 22°C was reached. The relative humidity was between 65% and 85%. The photoperiod “23 h on and 1 h off” was used. Feed and water were provided *ad libitum*, and no antibiotics or anticoccidial drugs were used during the first 10 days out of 28 days of the experiment.

All groups were subjected to identical environmental conditions of feeding and lighting and followed the preventive and health program recommended by the World Health Organization for broiler breeding.

### *E. tenell*a Oocyst isolation and infection protocol

The *E. tenella* oocysts used in this study were collected from the ceca samples of naturally infected chickens on the 7^th^ day after infection. These oocysts were sporulated, purified, and stored in 2.5% potassium dichromate at 25°C for 72 h and then stored at 4°C until use [31].

The identification of the sporulated oocyst species was based on morphological characteristics and morphometry as described by Al-Quraishy *et al*. [32], as well as the location of lesions. In addition, sporulated oocysts were passed twice in healthy chickens over 2 weeks of age before being used to activate the parasite and confirm the placement of the lesions. The oocysts were then collected, sporulated, washed, and adjusted to 1 × 10^4^/100 μL/bird [33].

### Experimental design and allocation of groups

Healthy experimental animals were randomly divided into six groups, each containing 10 birds, using a computer-generated randomization list to minimize selection bias. The overall study duration was 28 days.

- Group 1 (G1): Negative control (non-infected and non-treated)
- Group 2 (G2): Non-infected and treated by oral gavage with 100 μL of 0.133 μg/mL (ChSB-FeNPs) at 21 days old
- Group 3 (G3): Positive control: Infected and non-treated. A total of 1 × 10^4^ sporulated oocysts/100 μL distilled water was orally administered (oral gavage) to each experimental individual chicken from G3 to G6 at 14 days old [34].
- Group 4 (G4): Birds were challenged with *E. tenella* oocysts and treated by oral gavage with 100 μL of 0.133 μg/mL (ChSB-FeNPs) at 21 days old for 3 successive days
- Group 5 (G5): Birds were treated by oral gavage with 100 μL of 0.133 μg/mL of ChSB-FeNPs (prophylactic dose) at 11 days old (for 3 days) and then infected and challenged with *E. tenella* oocysts at 14 days old
- Group 6 (G6): Birds were challenged with *E. tenella* oocysts and treated (at 21 days old) with the anticoccidial drug, amprolium, administered through drinking water at the dosage recommended by the manufacturer (1.25 mL/L of water) for 3–5 days.

### Evaluation parameters (clinical signs, growth performance, mortality, oocyst count, and lesion score)

The weighed feed was offered *ad libitum* to all experimental broilers. The rejected feed was gathered and weighed before providing fresh feed the following morning.

Birds’ body weight gain (average body weight [ABW]) was estimated weekly on days 14, 21, and 28. The FCR was computed at the end of each week, and the final FCR was calculated at the completion of the experimental duration.

Survival rate: The Kaplan–Meier curve was plotted to estimate the survival rate; the number of surviving birds was recorded daily throughout the time of the experiment. Oocyst counts per gram of feces were recorded in each infected group using the McMaster technique on days 7 and 14 pi [35].

Lesion scoring of the ceca was carried out based on petechial hemorrhages, the thickness of the intestinal wall, and the severity of congestion. Scoring scale was (0) no changes, (+1) very mild changes, (+2) mild changes, (+3) moderate changes, and (+4) severe changes [36]. An independent professional observer who was unaware of the treatment groups conducted lesion assessment.

### Biochemical and histopathological analysis of the samples

At the end of the experiment, blood samples were collected for serum preparation. The liver enzyme levels (ALT, AST, and GGT,) LDL, and HDL were determined using specific kits (Roche Diagnostics, Switzerland). The birds were euthanized immediately after blood collection, and the samples of the cecal tissues were collected for histopathological examination and stained with hematoxylin and eosin for routine histological analysis [37].

### Statistical analysis

The data were coded and entered using the Statistical Package for Social Sciences version 26 (IBM Corp., Armonk, NY, USA). All values are expressed as mean ± standard deviation. The Shapiro-Wilk test and Levene’s test were used to check the assum-ptions of normality and homogeneity of variances before performing one-way analysis of variance. Comparisons between groups were performed using the *post hoc* Tukey test. Changes were considered significant if p < 0.05. All statistical analyses were performed using GraphPad Prism software version 10.2.3 for Mac (GraphPad Software, San Diego, CA, USA).

## RESULTS

### Nanocomposite characterization

#### FTIR

**Figure 2:**
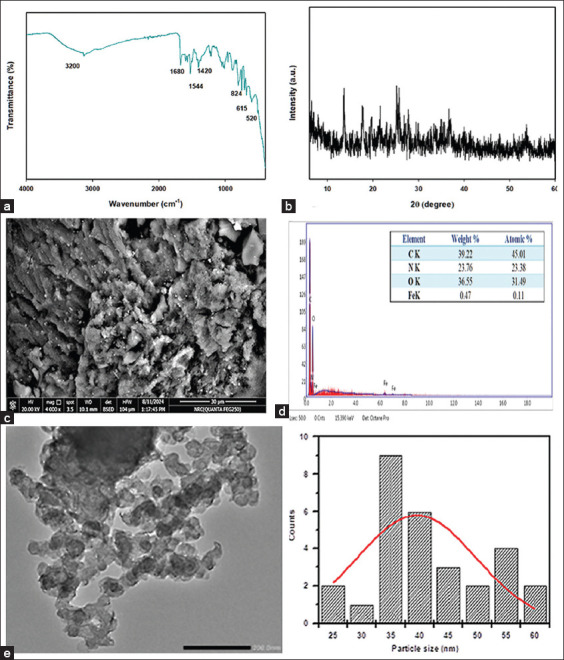
Characterization of chitosan Schiff base iron nanoparticles (ChSB-FeNPs). (a) Fourier-transform infrared spectroscopy analysis. (b) X-ray diffraction profile. (c) Scanning electron microscopy image. (d) Energy-dispersive X-ray. (e) Transmission electron microscope image.

FTIR analysis revealed characteristic bands of chitosan at 1420 cm^−1^ and 1155 cm^−1^, corresponding to stretching vibrations of the C–N amino groups and the O–C–O of the glucopyranoside ring, respectively. A strong band at 1680 cm^−1^ was attributed to the imine (C=N) vibration. Bands ranging from 1400 to 1500 cm^−1^ were indicative of aromatic C=C stretching, while the band at 1063 cm^−1^ suggested aromatic in-plane C–H bending. The appearance of bands at 1494 and 824 cm^−1^ confirmed the stretching of C=N and C–H bonds from the pyridine moiety. The broad band at 3200 cm^−1^ was attributed to hydroxyl and amino group vibrations, confirming the retention of chitosan functional groups (Figure 2a).

#### XRD

The XRD pattern displayed peaks at 2θ = 10.3° and 20.1° for pure chitosan. These peaks broadened on aldehyde modification, indicating reduced crystallinity due to structural alteration (Figure 2b).

#### SEM and EDX spectroscopy

SEM images revealed rough surface imperfec-tions in the Schiff base matrix (Figure 2c), a result of pyrazole-induced disruption of intrinsic polysaccha- ride hydrogen bonding. EDX spectra confirmed the presence of Fe, verifying the successful incorporation of Fe_2_O_3_ nanoparticles (Figure 2d).

#### TEM

TEM analysis showed semi-spherical Fe nanoparticles within the chitosan Schiff base matrix. The particles were uniformly distributed, with an average size of 39.5 nm (Figure 2e).

### Cytotoxicity evaluation

The compound and solvent exhibited complete cytotoxicity up to a 10^-^^3^ dilution, which was confirmed by cell deformation. A 10^-^^4^ dilution showed reduced toxicity (50%). Therefore, the 10^-^^5^ dilution (0.133 μg/mL) was determined to be safe for use.

### Clinical signs and mortality assessment

Birds in the infected untreated group (G3) developed signs of weakness, ruffled feathers, pallor, anorexia, hypothermic behavior, shaky movements, drooping wings, and dehydration. Diarrhea evolved from watery to bloody by the fifth day. While all infected groups showed bloody diarrhea, its severity was reduced in G4, G5, and G6.

**Figure 3:**
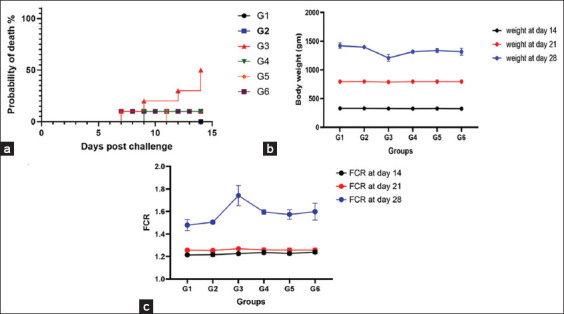
(a) Probability of death, (b) body weight gain, and (c) feed conversion ratios of chickens infected with *Eimeria tenella* and treated with ChSB-FeNPs. (G1) Negative control; (G2) negative non-infected, ChSB-FeNPs-treated; (G3) infected and non-treated; (G4) ChSB-FeNPs-treated after infection (curative dose); (G5) ChSB-FeNPs-treated before infection (prophylactic); and (G6) infected and treated with a conventional anticoccidial drug. ChSB-FeNPs=Chitosan Schiff base–iron nanocomposite.

No mortality was reported in G1 or G2. Notably, ChSB-FeNPs (G4) and amprolium (G6) significantly reduced mortality (p < 0.01) in infected broilers (Figure 3a). G3 showed the highest mortality. In contrast, prophylactic ChSB-FeNPs (G5) delayed the onset of mortality to day 11.

### Growth performance indicators

The lowest average body weight gain (ABW) was recorded in G3. Significant recovery in ABW was observed in G4 and G5 compared to G3 (p < 0.0001) by day 28 (Figure 3b). Amprolium (G6) showed no significant difference from G4 or G5 (p > 0.9999 and p = 0.4870, respectively).

Similarly, FCR improved significantly in G4 and G5 compared to G3 (p < 0.0001) (Figure 3c). No significant differences were found between G6 and G4 or G5 (p > 0.9999 and p = 0.5718, respectively), indicating the effectiveness of ChSB-FeNPs on growth performance.

### Oocyst shedding and lesion scoring

Oocyst counts were measured on days 7 and 14 post-infection (pi). G4, G5, and G6 had significantly reduced oocyst counts compared to G3 on both days (Table 1).

**Table 1:** Count of oocysts per gram (OPG) of faeces in *Eimeria tenella*-infected experimental chickens.

Group	No. of samples (n)	Day 7^th^ OPG±SD (% Reduction from G3)	No. of Samples (n)	Day 14^th^ OPG±SD (% Reductionfrom G3)	Effect Size (95%CI)
Group 1 (Uninfected)	10	-	10	-	-
Group 2 (Uninfected ChSB-FeNPs-treated)	10	-	10	-	-
Group 3 (Infected)	10	7700.9 ± 755.4^a^	7	4888.0 ± 351.3^b^	-
Group 4 (ChSB-FeNPs-treated, curative)	10	6556.1 ± 432.4^a^ (14.8)	9	3846.4 ± 311.46^b^ (21.3)	0.744 (728.10-1352.60)
Group 5 (ChSB-FeNPs-treated, prophylactic)	10	5657.6 ± 540.7^a^ (26.5)	9	2909.9 ± 227.8^b^ (40.4)	0.922 (1679.45-2318.42)
Group 6 (Amprolium-treated)	10	6024.7 ± 455.7^a^ (21.76)	9	2448.7 ± 233^b^ (49.9)	0.951 (2132.18-2689.19)

Means with different superscripts (a, b) inside a column are significantly different (*P*<0.05)

Lesion assessment revealed no lesions in G1 and G2. The infected control G3 showed moderate- to-severe lesions (+3 – +4), whereas the treated groups showed reduced severity. G5 exhibited the lowest lesion scores (+1 – +2), while G4 and G6 showed mild-to-moderate lesions (+1 –+3). No group displayed severe lesions (+4) post-treatment (Table 2).

**Table 2:** No. of chickens infected with *Eimeria tenella* and treated with ChSB-FeNPs, and amprolium according to lesion scores.

Group	0	+1	+2	+3	+4
Group 1	10	-	-	-	-
Group 2	10	-	-	-	-
Group 3	-	-	1	5	4
Group 4	-	2	5	3	-
Group 5	-	6	4	-	-
Group 6	-	2	4	4	-

(0) No changes, (+1) Very mild changes, (+2) Mild changes, (+3) Moderate changes, (+4) Severe changes, n = 10

### Serum biochemical parameters

#### Liver enzymes

**Figure 4:**
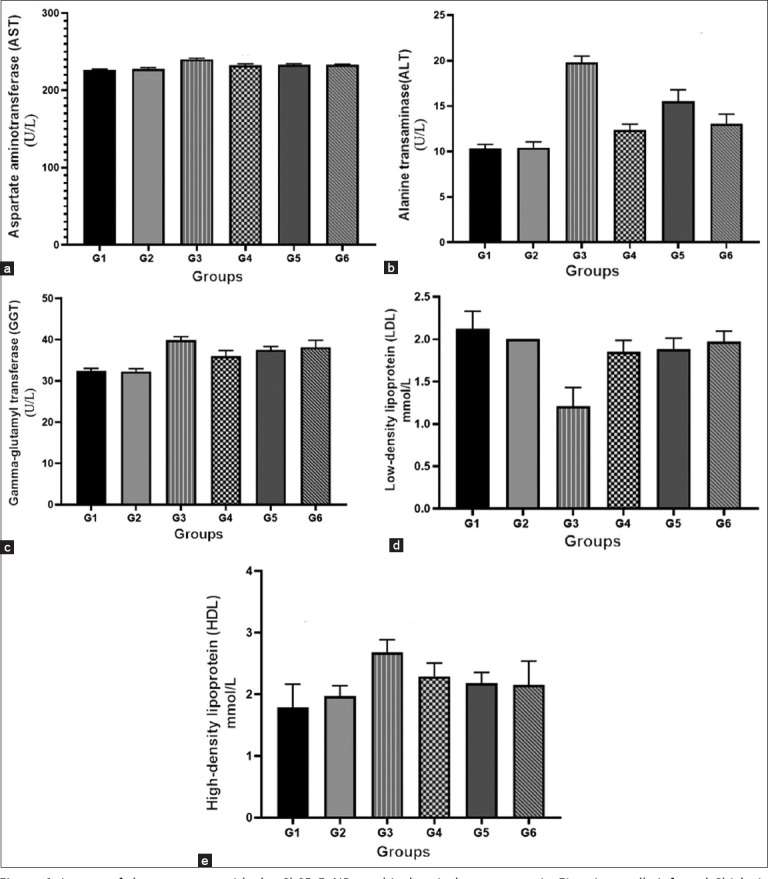
Impact of the treatment with the ChSB-FeNPs on biochemical parameters in Eimeria tenella-infected Chicks in different experimental groups, (a-c) liver enzymes; (a) alanine aminotransferase, (b) aspartate aminotransferase, and (c) gamma-glutamyl transferase. (d and e) Lipid profile; (d) low-density lipoprotein serum level, and (e) high-density lipoprotein serum level. Data are presented as Mean ± standard deviation. (G1) Negative control; (G2) negative non-infected, ChSB-FeNPs-treated; (G3) infected and non-treated; (G4) ChSB-FeNPs-treated after infection (curative dose); (G5) ChSB-FeNPs-treated before infection (prophylactic); and (G6) infected and treated with a conventional anticoccidial drug. ChSB-FeNPs=Chitosan Schiff base–iron nanocomposite.

G3 showed a significant increase in AST (240.20 ± 0.41 U/L) compared to G1 and G2. Treatment with ChSB-FeNPs (G4, G5) and amprolium (G6) significantly reduced AST levels (p < 0.0001) (Figure 4a). No differences were noted between G6 and G4 or G5 (p > 0.9999).

ALT levels were significantly higher in G3 (19.77 ± 0.22 U/L) versus G1 (10.30 ± 0.15 U/L) (Figure 4b). G4 exhibited the greatest reduction in ALT (12.35 ± 0.06 U/L), followed by G6 (13.04 ± 0.33 U/L) and G5 (15.50 ± 0.40 U/L) (p < 0.0001). No significant difference was found between G6 and G4 (p = 0.70).

GGT levels were elevated in G3 (39.84 ± 0.27 U/L) and significantly reduced by treatment in G4 (35.96 ± 0.43 U/L) and G5 (37.48 ± 0.26 U/L) (Figure 4c). G6 showed no difference compared to G5 but was significantly different from G4 (p = 0.0431).

#### Lipid profile

G3 had a significantly lower LDL (1.21 ± 0.06 mmol/L) than G1 (2.12 ± 0.06 mmol/L). G4 (1.85 ± 0.04 mmol/L), G5 (1.88 ± 0.04 mmol/L), and G6 (1.97 ± 0.03 mmol/L) demonstrated significant LDL recovery (p < 0.0001) (Figure 4d). No difference was observed between G6 and G4 or G5.

HDL was significantly elevated in G3 (2.68 ± 0.06 mmol/L) compared to G1 (1.79 ± 0.11 mmol/L) (Figure 4e). G6 showed the highest HDL reduction (2.15 ± 0.12 mmol/L), followed by G5 (2.18 ± 0.055 mmol/L) and G4 (2.29 ± 0.06 mmol/L). No significant differences were found among G4, G5, and G6.

### Histopathological findings

#### Normal controls

**Figure 5:**
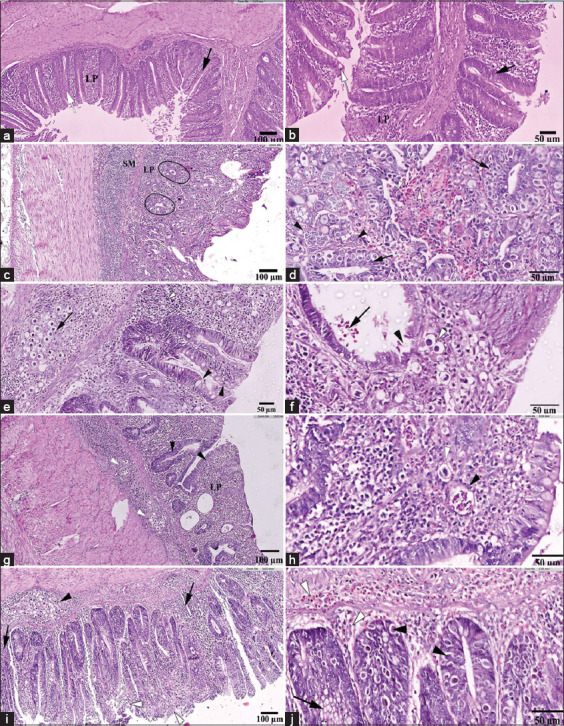
Photomicrographs of transverse section of ceca belonging to experimental groups of chickens. (a and b) Negative control and ChSB-FeNPs-treated groups, respectively; note the normal, healthy cecal tissue architecture with intact mucosal crypt epithelium (black arrow) with multiple goblet cells (white arrow) and lamina propria (LP) (hematoxylin and eosin [H&E]: 20×, scale bar: 50 μm). (c and d) Infected untreated group G3; (c) note degenerated and necrosed mucosal crypts’ epithelium (black circles) and marked mononuclear cellular infiltration in the LP and submucosa (SM) (H&E: 10×, scale bar: 100 μm). (d) Several developmental stages of *Eimeria* (black arrows) are identified, presence of clusters of schizonts containing merozoites (black arrowheads) and free banana-shaped merozoites (white arrowheads) are seen in the LP, (H&E: 40×, scale bar: 50μm). (e and f) Infected and ChSB-FeNPs-treated group G4; (e) note the hyperplasia of epithelial/goblet cells (black arrowheads) and a mild inflammatory cell infiltration (white arrowhead), besides a limited number of developing stages “degenerated oocytes” (black arrow) in the SM (H&E: 20×, scale bar: 50 μm). (f) Note the mild desquamation of the epithelial lining of the cecal crypts (black arrowhead) with some developing stages of parasite (white arrowhead). Free banana-shaped merozoites (black arrow) are detected in cecal crypts (H&E: 40×, scale bar: 50 μm). (g and h) ChSB-FeNPs-treated group then infected G5; (g) note very few developing stages of degenerated oocytes (white arrowheads) in the submucosal region and an observable improvement in both crypt epithelia (black arrowheads) and LP (H&E: 10×, scale bar: 100 μm). (h) Very few developing stages of degenerated oocytes (white arrowhead) are detected, as well as clusters of schizonts containing merozoites (black arrowhead) (H&E: 40×, scale bar: 50 μm). (i and j) Infected and amprolium-treated group G6; (i) marked developing stages of degenerated oocytes (black arrowhead) in the SM region, with moderate degree of damage to the cecal crypts (white arrowheads). In addition, infiltration of heterophilic and mononuclear cells (black arrows) in the LP and SM are noticed (H&E: 10×, scale bar: 100 μm). (j) Marked developing stages of degenerated oocytes (black arrowheads) in the epithelia of crypts, as well as free banana-shaped merozoites (white arrowheads) in the SM and LP, and hyperplasia in the cryptal goblet cells (H&E: 40×, scale bar: 50 μm). ChSB-FeNPs=Chitosan Schiff base–iron nanocomposite.

G1 displayed normal cecal histology with intact crypt epithelium and lamina propria (LP) (Figure 5a). G2, treated with ChSB-FeNPs alone, also showed no histopathological changes (Figure 5b).

#### Infected untreated control (G3)

Sections showed mucosal desquamation, epithelial degeneration, necrosis in crypts, and marked mononuclear infiltration. Developmental stages of *Eimeria*, including schizonts and gametocytes, were observed(Figures 5c and d).

#### Curative treatment (G4)

Tissue sections revealed mild epithelial desquamation and goblet cell hyperplasia. A few degenerated *Eimeria* stages and minor submucosal infiltration were present (Figures 5e and f).

#### Prophylactic treatment (G5)

Minimal degenerated oocysts were observed in the submucosa. There was notable epithelial and LP improvement (Figures 5g and h).

#### Amprolium treatment (G6)

Crypts exhibited moderate damage, cellular infiltration, and the presence of degenerated oocytes and goblet cell hyperplasia (Figures 5i and j).

## DISCUSSION

### Challenges in coccidiosis control

*Coccidiosis* in chickens is one of the most common and devastating parasitic illnesses in poultry production. It is caused by infection with parasites from the genus *Eimeria* and causes enormous econo-mic losses worldwide [38]. Traditional techniques to control and prevent coccidiosis include anti-coccidian drugs and live immunizations; however, these treatments raise problems regarding drug resis- tance, food security, production costs, and cross-species protection [39]. As a result, research into novel ways and ideas for coccidiosis management and prevention is critical.

### Need for novel alternatives

Drug resistance has emerged due to the use of anticoccidial medications, creating a significant challenge [40]. Consequently, a continuous need exists for effective and secure substitutes. In this regard, ChSB-FeNPs offer a promising avenue for research in this area.

### Characterization of the nanocomposite

#### FTIR and XRD analysis

FTIR spectroscopy revealed that characteristic chitosan bands (824, 1063, 1155, 1420, 1494, 1680, and 3200 cm^−1^) were observed in chitosan molecules in the studies by Hamed *et al*. [27] and Yurdakul and Badoğlu [41]. Furthermore, the bands at 615 and 520 cm^−1^ correspond to the Fe-O stretching band of Fe_2_O_3_, indicating their formation and strong interaction with chitosan Schiff base [42, 43].

The XRD pattern revealed that the characteristic peaks of pure chitosan at 2θ = 10.3° and 20.1° were broadened, indicating that the Schiff base exhibited a more amorphous nature compared to the established chitosan pattern [20, 44]. The disordered characteristics of the chitosan Schiff base can be ascribed to the disruption of chitosan’s regular structure caused by the introduction of the pyrazole aldehyde group. The observed peaks at 17.7°, 25.6°, 30.1°, 33.2°, 36.7°, 41.1°, 47.8°, 50.7°, and 53.8° correspond to the face-centered cubic phase reflections of the 202, 111, 220, 215, 311, 222, 400, 405, and 2212 lattice planes of Fe_2_O_3_ [43, 45].

#### SEM, EDX, and TEM findings

SEM/EDX analysis revealed that the addition of pyrazole to the polymeric chains of chitosan weakened the hydrogen bonds and intrinsic links between the polysaccharide chains within the polymeric matrix. The incorporation of the generated nanoparticles into the chitosan Schiff base matrix is supported by the presence of uniformly distributed round light particles. In addition, the examination using EDX spectroscopy revealed the presence of Fe.

TEM images showed semi-spherical Fe nanoparticles in the transparent central areas, with heavy dots in the chitosan Schiff base matrix. Image analysis produced a histogram of the size distribution and mean nanoparticle size, indicating that Fe_2_O_3_ averaged 39.5 nm.

### Therapeutic and prophylactic effects against E. tenella

#### Clinical outcomes and mortality

This study aimed to demonstrate the anticoccidial effects of ChSB-FeNPs as a curative dose and as a prophylactic treatment for *E. tenella* infection. As in many studies, the control infected group (G3) exhibited typical clinical signs associated with *E. tenella* challenge, including depression, weakness, pallor, bloody diarrhea, anorexia, and ruffled feathers. In addition, there was a significantly elevated cecal lesion score, and the mortality rate reached 50%.

Interestingly, chickens receiving ChSB-FeNPs as a prophylactic (G5) or as a curative therapy after infection (G4) exhibited reduced cecal lesion scores, lower mortality (10%), mild-to-moderate diarrhea, and decreasing numbers in oocysts per gram of feces (OPG) by day 14 compared with the positive control group (G3).

#### Immunomodulatory and antioxidant action

These findings can be attributed to the fact that chitosan and its Schiff-based derivatives are recognized as immunomodulators and antioxidants [2, 5]. Orally administered chitosan lowered inflammatory cytokines (tumor necrosis factor-alpha [TNF-α], transforming growth factor-beta) and boosted anti-inflammatory interleukin (IL)-10/IL-4, while reducing oxidative markers in *Eimeria*-infected gut tissue.

They can shift the immune response away from destructive inflammation (through nuclear factor-kappa B inhibition) and enhance antioxidant defenses (through Nrf2/HO-1), leading to the preservation of mucosal goblet cells (supporting barrier integrity) [34, 46].

Although there are no direct investigations of pyrazole-modified chitosan Schiff bases in *Eimeria*-infected chickens, the existing evidence suggests that similar mechanisms of action may be involved.

#### Nanocomposite synergy with Fe_2_O_3_

The addition of Fe_2_O_3_ nanoparticles to this composite in this investigation improved its efficacy as an anticoccidial alternative. The efficiency of Fe_2_O_3_ nanoparticles as an anti-protozoan agent has been studied either as a green synthesizer [47] or in combination with an antioxidant (e.g., quercetin) [48]. These studies demonstrated improvements in weight gain, blood biochemistry, suppression of pro-inflammatory genes (IL-1-βeta, inducible nitric oxide synthase, and cyclooxygenase-2), and enhanced immune responses. Therefore, incorporating these nanoparticles into the chitosan matrix may provide a synergistic defense against *Eimeria*.

### Growth performance and feed efficiency

Prophylactic G5, which received treatment before infection, exhibited a notably high ABW and enhanced FCR. Chitosan derivatives are broadly immunostimulatory [49–51], activating macrophages and T cells, skewing cytokines toward a Th2 response, supporting beneficial gut bacteria, and improving gut structure [46, 52, 53].

These benefits during the pre-infection period limit intestinal damage and enhance parasite clearance [54]. Blake *et al*. [55] stated that prophylactic doses of anticoccidial drugs are more efficient and cost-effective. However, further research is needed to optimize treatment protocols and fully elucidate the efficacy of this nanocomposite [55].

### Biochemical serum analysis

The biochemical serum examination of broilers infected with *E. tenella* revealed a marked rise in ALT, AST, and GGT levels, consistent with earlier studies by Mondal *et al*. [56], Deger *et al*. [57], and Athanasiou *et al*. [58]. Interestingly, the ChSB-FeNP-treated groups (G4 and G5) exhibited significantly reduced enzyme levels compared with the positive control (G3), indicating effective parasite suppression.

Chickens treated with amprolium (G6) also showed improved enzyme profiles, consistent with previous comparisons involving Brovitacoccid [59].

### Lipid profile assessment

Infested chickens (G3) exhibited decreased serum LDL and increased HDL levels compared to G1, likely due to anorexia and disruption of lipid metabolism [60–62]. In contrast, G4 and G5 showed reversed trends, suggesting better nutritional recover enhanced by chitosan derivatives [63]. Amprolium-treated birds (G6) exhibited even more favorable HDL and LDL levels, aligning with prior findings by Elbasuni *et al*. [61].

### Histopathological protection

Histological analysis of cecal tissues from G3 revealed severe epithelial necrosis and inflamma-tory infiltration, with several developmental stages of *Eimeria* evident [40, 64–66]. In contrast, G4 and G5 showed preserved mucosa, reduced inflammatory infiltration, and fewer *Eimeria* stages. This improvement may be attributed to the anti-inflammatory, anti-allergic, and antioxidant properties of the nanocomposite [67–70].

Chitosan promotes wound healing and immune cell activation and enhances gut barrier integrity through goblet cell preservation and mucin production [18, 71].

### Mechanisms of action: Role of molecular moieties

The nanodrug’s pyrazole core inhibits COX and LOX enzymes, reducing inflammation [72, 73]; its phenyl ring scavenges free radicals and alters host cell receptors [73–75]; and the pyridine ring suppresses TNF-α and IL-6 [76] while enhancing biological activity through hydrogen bonding and metal coordination [77, 78].

## CONCLUSION

This study demonstrated the promising anticoccidial efficacy of ChSB-FeNPs in broiler chickens experimentally infected with *E. tenella*. Both curative (G4) and prophylactic (G5) treatment regimens led to substantial reductions in cecal lesion scores, oocyst shedding, and mortality rates, decreasing mortality from 50% in untreated infected controls to 10% in treated groups. Clinical signs such as bloody diarrhea, weakness, and pallor were significantly alleviated, while body weight gain and FCR improved, especially in prophylactically treated birds.

Biochemical evaluations showed normalization of liver enzyme activities (ALT, AST, and GGT) and lipid profile markers (HDL and LDL), indicating reduced hepatic injury and systemic inflammation. These improvements were corroborated by histopathological findings, which revealed preserved cecal mucosa architecture, lower counts of *Eimeria* developmental stages, and reduced infiltration of inflammatory cells. Notably, the increased density of goblet cells suggested a protective effect on the intestinal mucosal barrier, likely due to the composite’s antioxidant and anti-inflammatory properties.

ChSB-FeNPs demonstrate a viable and natural alternative to synthetic anticoccidials such as amprolium, with dual prophylactic and therapeutic potential. Their integration into poultry management programs could reduce the frequency of drug use, mitigate the development of resistance, and enhance overall flock performance and food safety.

The study’s strengths include its comprehensive evaluation covering clinical, parasitological, biochemical, and histopathological parameters, as well as its comparative analysis of both treatment timings. However, the findings are based on controlled experimental settings, and field conditions may introduce additional variables. Moreover, molecular immune signaling pathways were not assessed, and the long-term safety and tissue residue profile of ChSB-FeNPs remain to be determined.

Future studies should focus on validating efficacy in commercial poultry operations, optimizing dosage and delivery formulations, and investigating molecular mechanisms of action, including immune gene expression and oxidative stress pathways. Exploring pharmacokinetics and establishing withdrawal periods will also be crucial for regulatory approval and the safe integration of this technology into poultry production systems.

In conclusion, ChSB-FeNPs represent a novel, multifunctional, and sustainable nanotherapeutic agent for the prevention and treatment of coccidiosis in poultry. Their ability to enhance immune responses, protect intestinal integrity, and improve health outcomes highlights their potential as a next-generation alternative to conventional anticoccidial drugs.

## AUTHORS’ CONTRIBUTIONS

HAT, MNA, and AHN: Designed the study and analyzed the data. AAH: Fabricated the nanocomposite drug. SJA: Collected samples, processed tissues, and carried out the histology. SJA, HAT, AAH, and AHN: Drafted the manuscript. All authors have read and approved the final version of the manuscript.

## DATA AVAILABILITY

All data generated or analyzed during this study are included in this published article.
